# A QCT-Based Nonsegmentation Finite Element Head Model for Studying Traumatic Brain Injury

**DOI:** 10.1155/2015/837585

**Published:** 2015-01-29

**Authors:** Zhaoyang Liang, Yunhua Luo

**Affiliations:** Department of Mechanical Engineering, University of Manitoba, Winnipeg, MB, Canada R3T 5V6

## Abstract

In the existing finite element head models (FEHMs) that are constructed from medical images, head tissues are usually segmented into a number of components according to the interior anatomical structure of the head. Each component is represented by a homogenous material model. There are a number of disadvantages in the segmentation-based finite element head models. Therefore, we developed a nonsegmentation finite element head model with pointwise-heterogeneous material properties and corroborated it by available experiment data. From the obtained results, it was found that although intracranial pressures predicted by the existing (piecewise-homogeneous) and the proposed (pointwise-heterogeneous) FEHM are very similar to each other, strain/stress levels in the head tissues are very different. The maximum peak strains/stresses predicted by the proposed FEHM are much higher than those by the existing FEHM, indicating that piecewise-homogeneous FEHM may have underestimated the stress/strain level induced by impact and thus may be inaccurate in predicting traumatic brain injuries.

## 1. Introduction

Due to the devastating consequences that may be caused by traumatic brain injury (TBI), considerable research has been devoted to understanding and to preventing TBI, for example, [1–8], among many others. Understanding of mechanical mechanisms involved is a prerequisite for preventing TBI and for designing more effective protective devices such as helmets. Due to its indispensable advantages over analytical modeling and physical experimentation, finite element modeling has become an increasingly important tool in uncovering mechanical mechanisms of TBI [2–6, 8]. However, the biofidelity of existing finite element head models (FEHM) is still to be improved. The biofidelity of FEHM includes mainly three aspects: geometric, material, and loading. Image-based finite element modeling provides an effective way for improving biofidelity of FEHM. Geometric biofidelity of FEHM has been greatly improved in recent years by generating finite element meshes from head medical images [2, 4, 5]. However, improvement in material and loading biofidelity as well as their implementation in FEHM has considerably lagged behind. In the following, the discussion is focused on improving the implementation of tissue material models in FEHM. In existing FEHM, head tissues are segmented into a number of anatomical components and each of them is represented by a homogeneous material model. Indeed, implementation of material biofidelity in FEHM can be improved by refining segmentation and by increasing the number of components. However, no matter how fine the tissues are segmented, tissue in each component is still heterogeneous. Therefore, heterogeneity of head tissues cannot be accurately described by existing FEHM. Furthermore, the quality of finite element mesh becomes poorer with segmentation refined, as smaller anatomical details are included. For example, the numerous sulci on the surface of brain tissues require complicated geometric surfaces to represent. To align with the complicated interfaces between the segmented tissues, finite elements there will inevitably be distorted. As it is well known, distorted finite elements will introduce spurious strains and stresses. Therefore, finite element analysis results obtained by a mesh consisting of distorted elements would be misleading if used in interpreting mechanical mechanisms involved in brain injuries or in establishing brain injury criterion based on stresses and strains. In this paper, to improve the implementation of tissue material models in FEHM, pointwise description of tissue heterogeneity is introduced; to improve quality of finite element mesh, head interior tissues are not segmented. Tissue material model is a very complex topic and includes many subaspects, for example, heterogeneity, anisotropy, and viscoelasticity. In this paper we mainly focus on how to fully describe heterogeneity of head tissues in FEHM.

## 2. QCT-Based Nonsegmentation Finite Element Head Model

**Figure 1:**
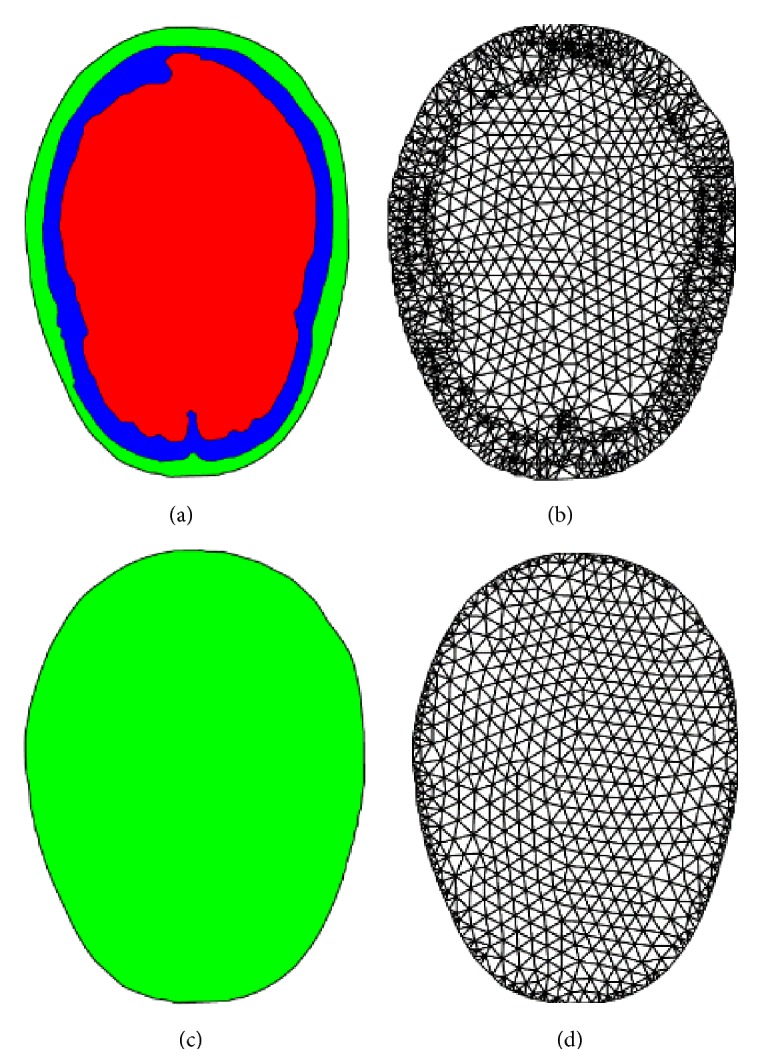
Existing FEHM: (a) geometric model, (b) FE mesh; the proposed FEHM: (c) geometric model, (d) FE mesh.

A sequence of cross-sectional images of tissues are produced in quantitative computed tomography (QCT) based on the amount of X-ray absorbed or attenuated by different tissues through which the X-ray travels [10]. The amount of X-ray absorbed by the tissue at a specific location is expressed as CT number and measured by Hounsfield Unit (HU) in the image. Denser tissues such as bones absorb more X-rays and thus have larger HU value, while water has lower density and thus smaller HU. Water is usually calibrated to have zero HU. Based on the above principle, QCT is utilized to measure bone mineral content or density and to characterize fluids and soft tissue lesions. On the other hand, if a set of QCT images and the corresponding scan settings are given, different tissues in the images can be distinguished by properly selecting a set of thresholds for the HU values, which has been implemented in medical image segmentation software such as Mimics [11] and Simpleware [12]. Therefore, the geometric and material information required for constructing a finite element model can be extracted from QCT images. The QCT images used in this study were obtained from the Health Science Centre located in Winnipeg, Canada, under a health research ethics approval. A number of FEHMs have been developed from medical images, for example, [2–6], among others. In the existing FEHM, a set of HU thresholds are used to identify the interfaces of different head tissues and a geometric model is constructed. Each anatomical component in the head is represented by a geometric region and a homogeneous material. However, each component tissue is still heterogeneous. Therefore, inhomogeneity of head tissues is not fully represented in the existing FEHM. Furthermore, due to the complexity in the head anatomical structure, the obtained geometrical model is usually very complicated, depending on the anatomical details represented in the finite element model; the quality of the finite element mesh is thus poor. To resolve the above issues, an alternative FEHM is proposed in the following. In the proposed FEHM, HU thresholds are only used to determine the material model adopted at a specific location, for example, at the Gaussian integration points in calculating of element stiffness matrices. The geometric model of the head is constructed from the outmost surface extracted from QCT images. As no interior tissue interface is represented in the geometric model, the model is much simpler and the finite element mesh also has much higher quality. The difference between the proposed and existing FEHM can be demonstrated using a two-dimensional case shown in Figure 1. In the illustration, the head tissues are simplified into three components, that is, the skull, the cerebrospinal fluid (CSF), and the brain, as considered in the literature [8]. The segmentation could be further refined to include more anatomical details such as ventricles, cortical and cancellous layers in the skull, and even neurons and axons. The geometric model would definitely become more complicated. For the proposed FEHM, the constructed geometric model is shown in Figure 1(c).

With a similar mesh density, the geometric model in Figure 1(a) requires 2797 elements to delineate the interior interfaces and there also exist a large number of distorted finite elements, which mainly locate in the vicinity of tissue interfaces, while the geometric model in Figure 1(c) can be represented by a high-quality finite element mesh that has only 1310 elements.

**Figure 2:**
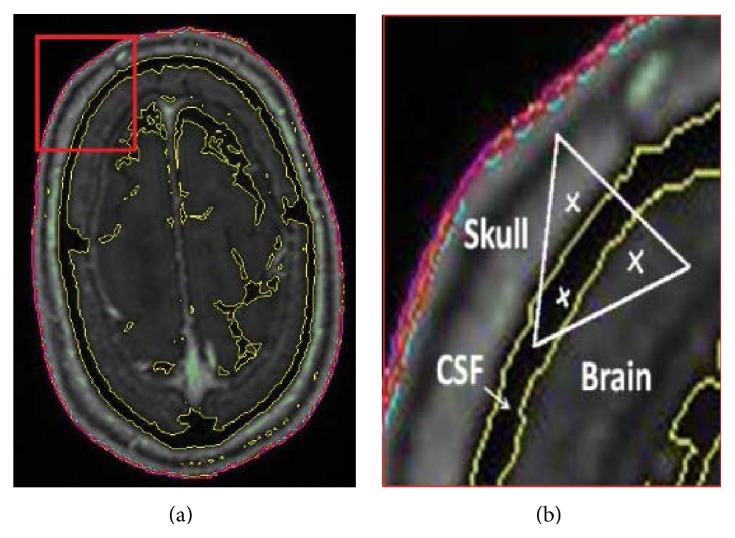
(a) QCT image and a concerned region zoomed in (b); (b) Gaussian quadrature points located in three different tissues.

Assignment of material properties in the proposed FEHM is also different from existing FEHM. In existing FEHM, each tissue component is treated as a homogeneous material and the same set of material properties is assigned to the whole component [8]. For example, for the head model shown in Figure 1(a), three sets of material properties are assigned, respectively, to the skull, the cerebrospinal fluid, and the brain. In the proposed FEHM, head tissues are treated as pointwise-heterogeneous material and material properties are correlated to HU values by empirical functions, which will be described in detail in the next section. One representative scenario is illustrated in Figure 2 using a slice of QCT image, where a triangle element spans over three different tissues. If the three-point Gaussian quadrature rule is used in calculation of the element stiffness matrix, the locations of the quadrature points are indicated by symbol “*x*” in Figure 2(b). For each Gaussian quadrature point, its spatial coordinates are known and its HU value can be mapped from the QCT images. The HU value is then used to determine the type of the tissue and the adopted material model at the point according to Table 1, where *H*_0_, *H*_1_, *H*_2_, and *H*_3_ are a set of HU thresholds that are used to segment the cerebrospinal fluid (CSF), the brain soft tissue, and the skull. The thresholds can be determined either by experimental calibration or by adopting those values used in Mimics [11] or Simpleware [12]. In this study, the following HU thresholds were taken from Mimics [11]: *H*_0_ = 0, *H*_1_ = 55, *H*_2_ = 755, and *H*_3_ = 1955. For each material model, HU values are correlated to tissue mass density and then to tissue mechanical properties by empirical functions.

## 3. Correlations of Tissue HU Value, Mass Density, and Mechanical Property

Although the proposed FEHM is in principle able to include all small anatomical structures of the head, the head was represented by only three components, that is, the skull, the brain, and the cerebrospinal fluid, due to the limited resolution of the obtained QCT images and the difficulty in obtaining all the required material properties for all the small anatomical components. In the following, how tissue HU values are first correlated to mass densities and how mass densities are then correlated to mechanical properties, for the cerebrospinal fluid, the skull, and the brain, respectively, are described.

The cerebrospinal fluid is nearly an incompressible fluid (CSF) [1]. Therefore, it is reasonable to consider CSF as a homogeneous material. Its equivalent Young's modulus *E* = 0.5 MPa, Poisson's ratio *ν* = 0.4998, and mass density *ρ* = 1.045 g/cm^3^ were taken from the literature [1].

For the skull, the correlations were established by extensive experimental studies using animal and human cadaveric bones [13]. Skull bone mineral density (*ρ*, g/cm^3^) is correlated to HU value (*H*) by the following linear function:

(1) $$\begin{array}{c} \rho =a_{0}+b_{0}H, \end{array}$$

where *a*_0_ and *b*_0_ are coefficients determined by experimental data using the linear regression. Bone mineral density measured by QCT has a high correlation with ash density [14]. Elasticity modulus (*E*) of skull bone is obtained from mass density (*ρ*) by exponential function [9],

(2) $$\begin{array}{c} E=a_{1}\rho^{b_{1}}, \end{array}$$

where coefficients *a*_1_ and *b*_1_ are determined by experimental data.

It was found by experiment studies [15, 16] that mass density of soft tissue (*γ*) is related to HU also by a linear function:

(3) $$\begin{array}{c} \gamma =c_{0}+d_{0}H, \end{array}$$

where *c*_0_ and *d*_0_ are experimentally determined coefficients.

However, very little research has been reported on correlation between mechanical property of soft tissue and its mass density. In this study, the following correlation was assumed for soft tissues, which is similar to that of bones:

(4) $$\begin{array}{c} E=c_{1}\gamma^{d_{1}}. \end{array}$$

The coefficients *c*_1_ and *d*_1_ were determined in the following way. Three samples of brain tissue were taken from QCT image in different region. The samples have the same dimensions as those used in the experiment [1, 13]. Mass density distribution in the samples was determined using (3). The coefficients in (4) were determined by minimizing the following function with respect to *c*_1_ and *d*_1_:

(5) $$\begin{array}{c} \varsigma =\overset{3}{\underset{i=1}{\sum }}{\left({\overset{-}{E}}_{i}-E^{*}\right)}^{2}, \end{array}$$

where *E* ^*^ is the elasticity modulus measured by experiment; ${\overset{-}{E}}_{i}$ is the average elasticity modulus of sample *i* that was calculated as

(6) $$\begin{array}{c} {\overset{-}{E}}_{i}=\frac{\sum_{j=1}^{n_{i}}\left(c_{1}\gamma_{j}^{d_{1}}\right)V_{j}}{\sum_{j=1}^{n_{i}}V_{j}}, \end{array}$$

where *n*_*i*_ is the number of voxels in sample *i*; *V*_*j*_ is the volume of voxel *j*, which is the same for all voxels.

**Figure 3:**
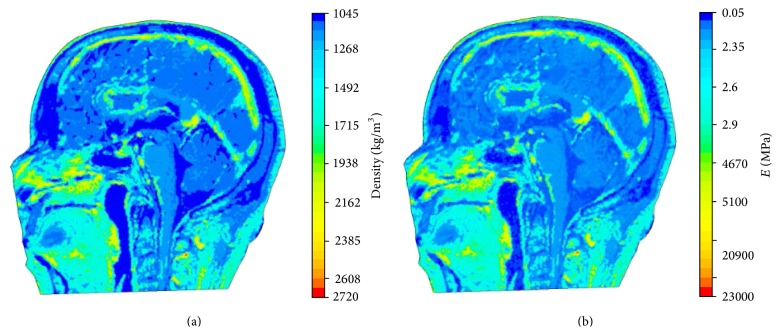
(a) Mass density distribution; (b) Young's modulus distribution.

**Figure 4:**
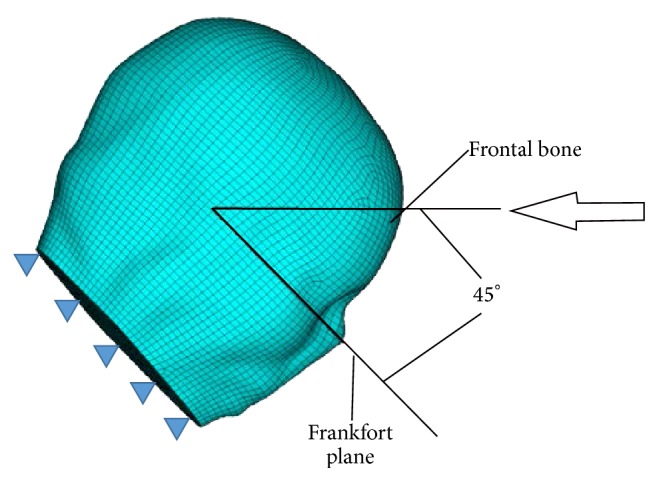
Head impact model.

**Figure 5:**
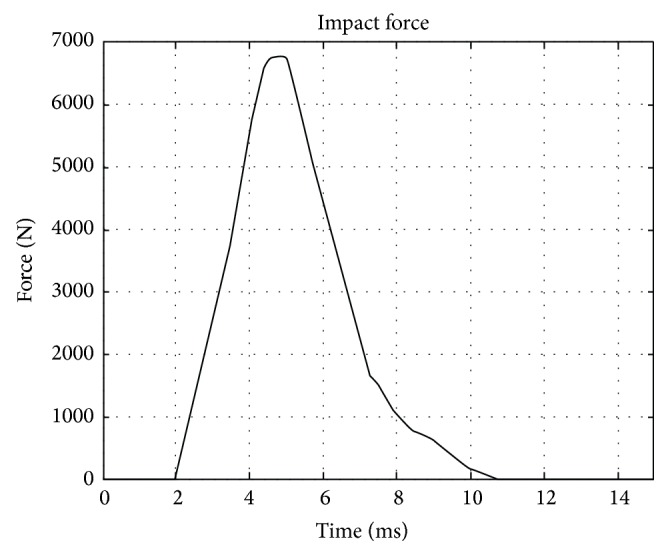
Impact force used in experiment and simulation.

The resulting distributions of mass density and elasticity modulus over the middle sagittal plane are displayed in Figures 3(a) and 3(b), respectively. It can be seen that pointwise heterogeneity of head tissues is fully represented in the proposed FEHM. To investigate the effect of considering pointwise heterogeneity on stress and strain level in the brain tissues, virtual impact tests were conducted to simulate the cadaver impact tests by Nahum et al. [17]. The setup of the virtual impact test is shown in Figure 4. The impact force and constraint information required in the simulations were extracted from [17]. The impact force retrieved from [17] and displayed in Figure 5 was applied normally at the middle of the forehead. The neck was completely constrained as the head was a part of the body in the experiment and the duration of the impact force was very short. QCT images were obtained from the Winnipeg Health Science Centre to construct FEHM. The subject was a deceased male of 56 years old. The cadaver was scanned using a clinical CT scanner (Siemens, CPS Innovations, USA) with the following acquisition parameters: 120 kVp, 244 mAs, resolution 512 × 512, slice distance 3 mm, and pixel spacing 0.53571 mm × 0.53571 mm. Two FEHMs were constructed from the same set of QCT images. One FEHM has piecewise-homogeneous material description; the other has pointwise-heterogeneous material distribution. In both FEHM, 8-node hexahedral elements were used. Upon convergence, the piecewise-homogeneous model had 358,927 elements; the pointwise-heterogeneous model had 278,816 elements. The coefficients in the empirical correlation functions and adopted in this study are listed in Table 2 [17].

## 4. Results and Discussions

**Figure 6:**
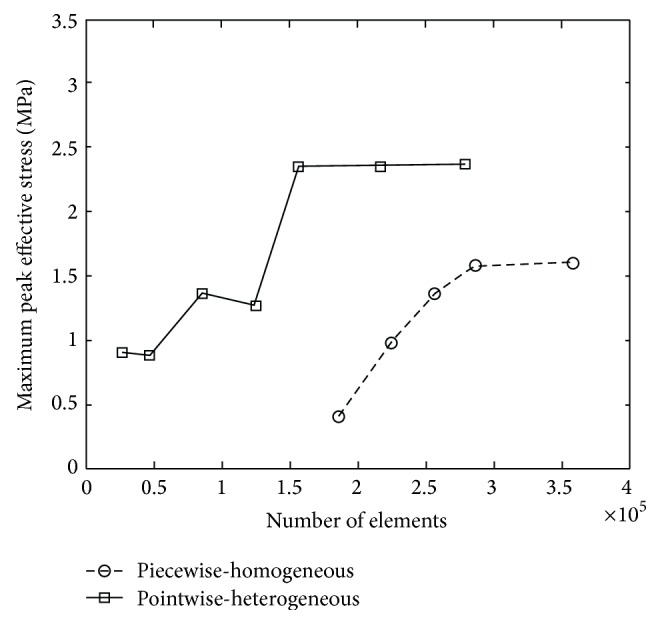
Convergence of maximum peak effective stress.

**Figure 7:**
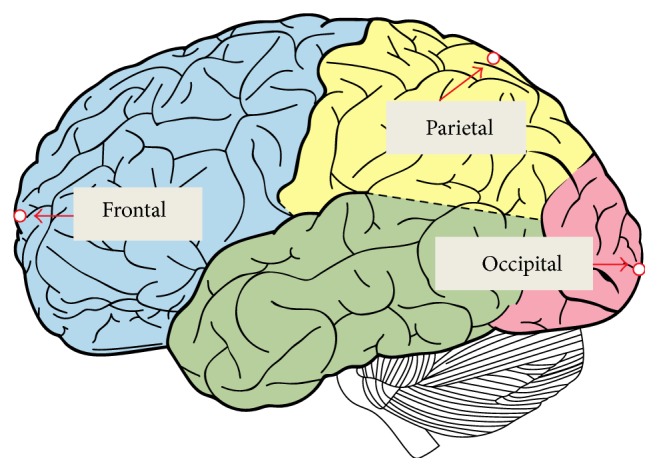
(a) Finite element mesh without interior segmentation; (b) locations of computed intracranial pressure.

**Figure 8:**
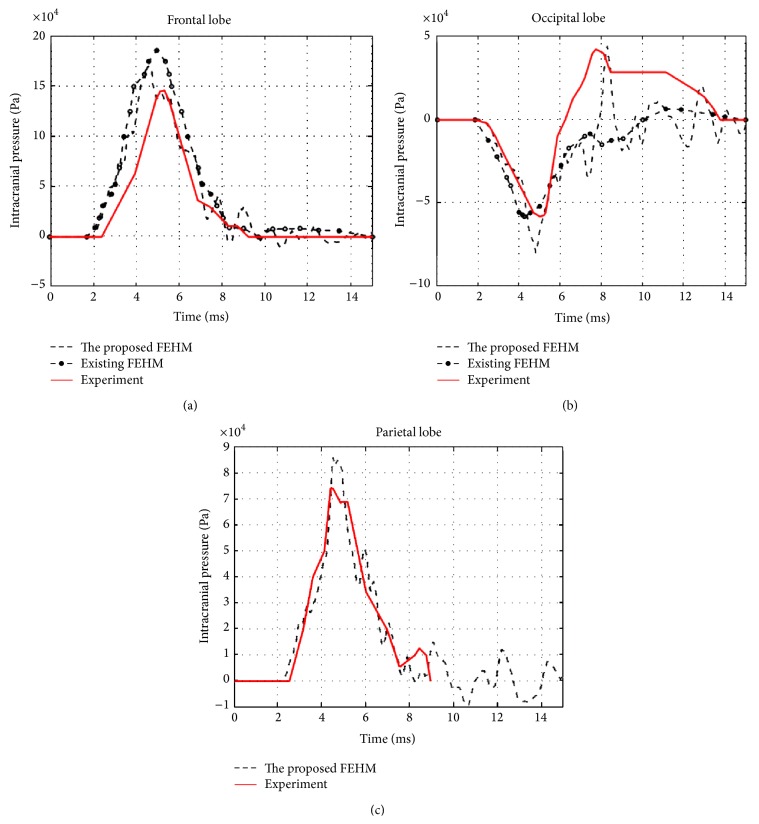
(a) Frontal pressure; (b) occipital pressure; (c) parietal pressure.

**Figure 9:**
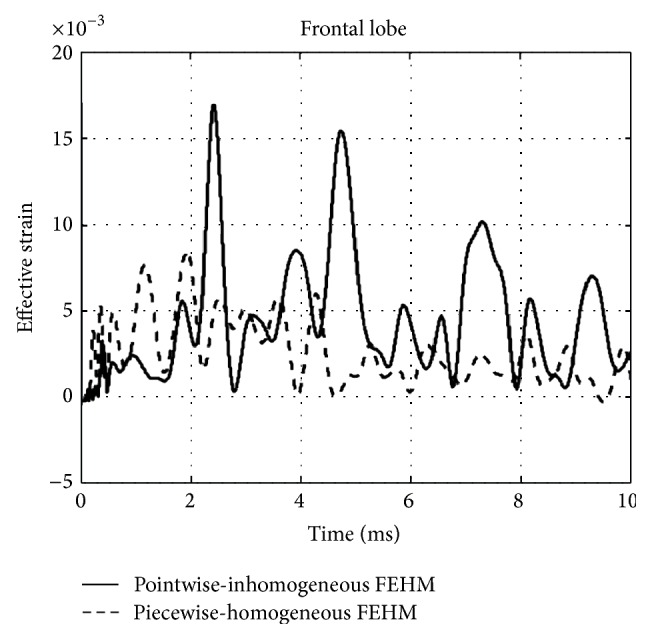
Effective strain at a point located in the frontal lobe.

The convergences of maximum peak effective stresses in the two FEHMs are plotted in Figure 6. Intracranial pressure, maximum peak effective stress, and maximum peak effective strain have been proposed in the literature as brain injury criteria [1, 3]. Therefore, they were computed and compared with the available experimental data and existing FEHM. Intracranial pressures at the locations shown in Figure 7, that is, at the frontal, the occipital, and the parietal lobe, were computed. The obtained results are plotted in Figures 8(a), 8(b), and 8(c), where experimental data from [17] and simulation results obtained by Chen and Ostoja-Starzewski [18] are also displayed for comparison. The maximum peak intracranial pressure and the maximum peak effective stresses/strains predicted by the piecewise-homogeneous and the pointwise-heterogeneous FEHM are listed in Table 3. It should be noted that the maximum peak effective stress and the maximum peak effective strain may not locate at the same point. The time history of effective strain at a point located in the frontal lobe is shown in Figure 9.

From Figure 6, it can been seen that the convergence of the piecewise-homogeneous model is monotonic, while oscillations can be observed in the convergence of the pointwise-heterogeneous model, which has been caused by the heterogeneity in the head tissues. Upon convergence, the piecewise-homogeneous model required more elements, mainly to represent the complex interfaces between the head tissues. From Figure 8 it can be seen that the intracranial pressures predicted by the proposed pointwise-heterogeneous FEHM are in a reasonable agreement with the experimental data. The differences between the simulated and the experimental intracranial pressures may have been caused by individual anatomical differences in the subjects used in the experiment and in the simulation. The oscillations in the intracranial pressure have been caused by considering tissue heterogeneity. The results in Table 3 indicated that there is no significant difference between the piecewise-homogeneous and the pointwise-heterogeneous FEHMs in predicting intracranial pressure. However, there exist significant differences in the maximum peak stresses/strains predicted by the two models. The reason may be that, in the piecewise-homogeneous FEHM, averaged material properties have been adopted for a whole tissue component and the strength of the weaker tissues has thus been overestimated.

## 5. Conclusions

The proposed pointwise-heterogeneous FEHM is able to more truthfully describe heterogeneity in tissue properties. As there is no need to represent the interior tissue interfaces, the pointwise-heterogeneous FEHM is also computationally more efficient. Although the obtained results suggest that there is no significant difference in the intracranial pressures predicted by the piecewise-homogeneous and the pointwise-heterogeneous FEHM, the piecewise-homogeneous FEHM may have significantly underestimated the stress/strain level in the brain tissue induced by impact. Therefore, if stress or strain is used as brain injury criterion, the piecewise-homogeneous FEHM may not be reliable in predicting brain injury.

## Figures and Tables

**Table 1:** HU range for different head tissues.

HU range	Head tissue
*H*_0_ ≤ *H* < *H*_1_	Cerebrospinal fluid (CSF)
*H*_1_ ≤ *H* < *H*_2_	Brain soft tissue
*H*_2_ ≤ *H* ≤ *H*_3_	Skull

**Table 2:** Correlation coefficients adopted in this study [9].

*a*_0_	1.3036	*c*_0_	1.0494
*b*_0_	4.4236 × 10^−4^	*d*_0_	1.9531 × 10^−4^
*a*_1_	2.954	*c*_1_	1.835
*b*_1_	2.41	*d*_1_	2.72

**Table 3:** Comparison of piecewise-homogeneous and pointwise-heterogeneous FEHM.

	Piecewise-homogeneous FEHM	Pointwise-heterogeneous FEHM
Maximum peak intracranial pressure	0.16 (MPa)	0.18 (MPa)
Maximum peak effective strain	0.0088	0.026
Maximum peak effective stress	1.6 (MPa)	2.37 (MPa)
Maximum peak shear strain	0.0074	0.017
Maximum peak shear stress	0.41 (MPa)	1.03 (MPa)
